# American Medical Association

**Published:** 1886-03-20

**Authors:** 


AMERICAN MEDICAL ASSOCIATION.—It has been announced that the Association will meet in St. Louis, on the 4th of May, and the agents of the various Railroads terminating in that city have published that tickets to the meeting of the Association will be sold at the rate of one-third full fare for the round trip. Similar arrangements have been also entered into by the Steamboat lines.

